# Positive implicit followership theories relate to followers' innovative behavior via future work self salience and job crafting

**DOI:** 10.3389/fpsyg.2026.1935850

**Published:** 2026-10-19

**Authors:** Yanlei Yang

**Affiliations:** 1 School of Business, Zhengzhou University of Industrial Technology, Zhengzhou, China; 2 Faculty of Management and Economics, Universiti Pendidikan Sultan Idris, Tanjong Malim, Malaysia

**Keywords:** followership, future work self salience, innovative behavior, job crafting, positive implicit followership theories

## Abstract

Employee innovative behavior is a key contributor to organizational adaptability and competitiveness, and understanding its psychological antecedents remains a central concern for both scholars and practitioners. Although implicit followership theories (IFTs)—the cognitive prototypes that individuals hold about what followers are like—have been linked to workplace outcomes, most prior work has adopted a leader-centric view and has examined IFTs in relation to innovation through relational accounts such as leader–member exchange. Adopting a follower-centric perspective and integrating possible-selves theory with the model of proactive motivation, the present study proposes that followers‘ own positive IFTs are indirectly associated with their innovative behavior via two self-concept-based, agentic intervening variables, future work self salience and job crafting. The model was estimated with data from a three-wave, time-lagged design with matched employee–supervisor dyads of knowledge workers in Chinese enterprises (*N* = 258). Positive IFTs, the two intervening variables, and supervisor-rated innovative behavior were measured at separate waves; the two intervening variables were measured concurrently. Confirmatory factor analysis supported the discriminant validity of the focal constructs; the positive IFT measure was adapted to a self-referent framing, and its construct validity was not independently established. Regression-based path analysis showed that positive IFTs were positively related to innovative behavior, and bootstrap analyses indicated a significant indirect association between positive IFTs and innovative behavior via future work self salience and job crafting, while the direct association remained significant. These findings extend the followership literature by indicating that followers' own positive prototypes are associated with innovation, by examining self-concept-based intervening variables that complement established relational accounts, and by suggesting that positive role-related beliefs are associated with self-relevant cognitions. The design does not permit causal inference or inference about the temporal ordering of the intervening variables. Theoretical and practical implications, limitations, and directions for future research are discussed.

## 1 Introduction

Under conditions of rapid technological change, shifting customer demands, and intensifying competition, the capacity of organizations to innovate depends substantially on the innovative behavior of their individual members. Employee innovative behavior—defined as the intentional generation, promotion, and realization of new ideas, products, processes, or procedures within a work role (Scott and Bruce, 1994)—constitutes the individual-level basis of organizational innovation and renewal. Because innovative behavior is discretionary and extends beyond formally prescribed tasks, it cannot be required; instead, it emerges when employees are both motivated and psychologically equipped to depart from routine and to invest effort in novelty. Consequently, a substantial body of research has sought to identify the dispositional, motivational, relational, and contextual factors that encourage employees to behave innovatively, and recent studies continue to refine understanding of the proximal psychological pathways that link such factors to innovative behavior (Song and Jo, 2023).

One increasingly influential approach to understanding workplace behavior focuses not on what leaders or followers objectively do, but on the cognitive structures through which organizational members interpret the roles of leader and follower. Implicit followership theories (IFTs) refer to the personal assumptions, or prototypes, that individuals hold about the traits and behaviors that characterize followers (Sy, 2010). In parallel with implicit leadership theories, individuals hold implicit theories about what followers are like; these prototypes are cognitively efficient, are activated automatically, and guide expectations, evaluations, and behavior in ways that are relevant for individuals and organizations. Since its systematic articulation, IFT research has linked these prototypes to workplace perceptions and behaviors, although the outcomes examined vary across contexts (Sy, 2010). Recent review work likewise identifies follower implicit followership as an emerging, follower-centered research area (Matshoba-Ramuedzisi et al., 2022).

Research on the antecedents of innovative behavior has developed several lines of explanation. Dispositional accounts point to traits such as proactive personality and openness to experience; motivational accounts emphasize intrinsic motivation, creative self-efficacy, and goal orientation; relational and leadership accounts highlight the role of supervisors, teams, and exchange relationships; and contextual accounts stress climate, resources, and job design. Across these streams, a consistent finding is that innovation is most likely when employees possess both the motivation to initiate change and the psychological and situational resources to sustain it. What remains comparatively underdeveloped, however, is an account rooted in employees' beliefs about their own work role and in the self-relevant cognitions associated with those beliefs. The present study addresses this gap by examining whether a role belief is indirectly associated with supervisor-rated innovative behavior via a future-oriented self-representation and a proactive work behavior. The present article does not attempt to review each stream exhaustively; the most relevant prior evidence for the current model concerns relational pathways to innovation and job-design and job-crafting pathways (Liang et al., 2022; Miao et al., 2022; Song and Jo, 2023).

Nevertheless, the way in which the field has studied IFTs and, more broadly, followership, indicates two gaps that the present study addresses. First, research on followership has historically been dominated by a leader-centric orientation. The prevailing question has typically been how leaders' beliefs about followers—or how leaders' behaviors toward followers—relate to follower outcomes, whereas the perspective of followers themselves, and their agentic contributions to the workplace, has received comparatively limited attention (Epitropaki et al., 2013; Uhl-Bien et al., 2014). Recent followership theorizing increasingly treats followership as an active, relational process in which followers may exercise agency (Uhl-Bien et al., 2014). Consistent with this perspective, the present study shifts the focal predictor from leaders' IFTs to followers' own positive IFTs—that is, the extent to which employees personally endorse a positive followership prototype characterized by being industrious, enthusiastic, and a good citizen. A follower-centric account is theoretically important because employees' beliefs about the follower role are self-relevant cognitions that may plausibly be associated with how they see themselves and with how they act. A recent systematic review further emphasizes the active contribution of followers while calling for clearer conceptual boundaries in empirical followership research (Ribbat et al., 2024).

Second, even within the emerging work that links IFTs to innovation-related outcomes, the dominant explanatory logic has emphasized leaders' IFTs and relational, exchange-based accounts. A representative example indicates that leaders' positive IFTs relate to employees' innovative behavior through leader–member exchange and psychological empowerment, an account centered on the quality of the leader–follower relationship (Liang et al., 2022). Although such relational accounts are valuable and well supported, they leave open a theoretically distinct possibility: that followers' own positive prototypes may be associated with innovation via internal, self-concept-based agentic variables that do not depend on the leader–follower relationship. If followers who endorse a positive followership prototype report greater salience of their hoped-for working future, and if that self-view is linked to proactive reshaping of work, then innovation may be associated with variables that are internal to the follower. To date, this internal agentic account has been underexplored, and the intervening role of self-concept and proactive work behavior in the IFT–innovation relationship has rarely been tested directly. Because direct evidence linking followers' own positive IFTs to innovation remains limited, this internal pathway is presented as a theoretically grounded research question rather than as an established empirical mechanism.

To address these gaps, the present study integrates two complementary theoretical frameworks. Possible-selves theory holds that individuals maintain cognitive representations of what they might, hope to, or fear becoming, and that these future self-representations may guide and energize present behavior (Markus and Nurius, 1986). The model of proactive motivation specifies how such representations are linked to agentic action by distinguishing “can-do,” “reason-to,” and “energized-to” motivational states (Parker et al., 2010). Bringing these frameworks together, the present study proposes that followers' positive IFTs are indirectly associated with innovative behavior via future work self salience and job crafting. Specifically, endorsing a positive followership prototype is expected to be associated with greater salience of employees' hoped-for future work selves; a salient future work self may provide a “reason-to” motivational basis for job crafting; and job crafting is associated with resources, challenges, and meaning relevant to innovative behavior. Taken together, these three associations imply an indirect association between positive followership beliefs and innovative behavior. Within this framework, job crafting is the job-directed form of proactivity through which a future-oriented self-concept may be expressed in current work by changing tasks, relationships, and demands (Tims et al., 2012; Miao et al., 2022; Song and Jo, 2023). Self-categorization theory is drawn on only as background for the self-relevance of followership beliefs (see Theory and hypotheses); follower identification was not measured, and no identification process is tested.

The present study offers three contributions. First, by positioning followers' own positive IFTs as a construct associated with innovative behavior, it advances a follower-centric account of workplace innovation that complements the predominantly leader-centered followership literature and responds to calls for research on proactive and innovative behavior from a followership perspective (Yalçinyigit and Karaçay, 2025). Second, by examining future work self salience and job crafting as intervening variables, it develops an internal, self-concept-based agentic account that complements the relational pathways emphasized in prior IFT research, and thereby contributes to understanding of how positive prototypes are associated with innovation. Third, by employing a three-wave, time-lagged design with supervisor-rated innovative behavior, it provides a methodologically stronger test that reduces common method concerns, although the two intervening variables were measured concurrently and their temporal ordering is not tested. Collectively, these contributions add to understanding of when and how followers' beliefs about the follower role are associated with innovation-related behavior.

## 2 Theory and hypotheses

### 2.1 Theoretical background: possible selves and proactive motivation

The theoretical account rests on two complementary frameworks. Possible-selves theory conceptualizes the self-concept as encompassing not only representations of the current self but also representations of future selves—selves that individuals hope to become, expect to become, or are afraid of becoming (Markus and Nurius, 1986). Possible selves may serve two central functions: they provide direction by specifying valued end states toward which behavior can be oriented, and they provide motivation for pursuing those end states. The motivational relevance of a possible self depends on its salience and clarity; a clearly elaborated, accessible future self is more likely to be associated with sustained, goal-directed action than a vague or peripheral one. Applied to the domain of work, this reasoning implies that the way employees envision their future working selves should be associated with the proactive investments they make in the present.

The model of proactive motivation complements possible-selves theory by articulating the motivational pathways through which future-oriented cognitions are linked to agentic behavior (Parker et al., 2010). This model distinguishes three proximal motivational states: “can-do” motivation, which reflects perceptions of capability and control; “reason-to” motivation, which reflects the reasons, meaning, or purpose that make proactivity worthwhile; and “energized-to” motivation, which reflects the activated affective states associated with action. Among these, “reason-to” motivation is especially pertinent to the present model, because future work self salience provides employees with a reason to act proactively—namely, to close the gap between who they are now and who they aspire to become at work. Integrating the two frameworks yields the organizing framework of the present study: role-relevant beliefs are associated with a salient future self-image; a salient future self-image may supply a motivational reason to engage in proactive behavior; and proactive behavior is associated with valued outcomes such as innovation. The framework is applied from a follower-centric standpoint, with followers' positive IFTs as the belief at the start of the proposed model. The associations examined are grounded in possible selves and proactive motivation rather than in interpersonal expectation effects.

Two features of this framework should be noted. The first concerns direction: possible selves and proactive motivation are future-oriented and self-initiated, which makes them theoretically relevant to innovation, itself a forward-looking, change-oriented activity. The second concerns the arrangement of the intervening variables: the two frameworks describe cognition as a basis for motivation and motivation as a basis for proactive action, and future work self salience is therefore entered before job crafting in the model. Because the two variables were measured at the same wave, this arrangement is not tested in the present study. The hypotheses below specify associations between adjacent constructs in the framework.

The two frameworks can be contrasted with two alternative accounts. A purely relational account, such as one centered on leader–member exchange, would locate the focal source of innovation-related behavior in the quality of the dyadic relationship and would therefore predict that the association between IFTs and outcomes depends on how the leader responds to the follower. A purely trait-based account would treat innovation as a relatively stable expression of individual differences and would assign a limited role to malleable, belief-related variables. The present integrated account differs from both. By grounding the process in the self-concept and in proactive motivation, it treats innovation as associated with agentic cognitions and behaviors of the follower, which are themselves associated with beliefs that are susceptible to framing and development. This positioning is consistent with the follower-centric orientation of the study and clarifies why an internal set of associations is expected to complement, rather than duplicate, the relational pathways documented in prior work.

A further body of theory provides background for treating followership beliefs as self-relevant. Self-categorization theory holds that when individuals categorize themselves as members of a social category, they tend to assimilate the prototypical attributes of that category into their self-definition (Turner et al., 1987; Hogg and Terry, 2000). Applied to followership, this reasoning implies that employees who identify as followers may incorporate the attributes of their followership prototype into their self-concept. Follower identification was not measured in the present study, and this account is presented as background only; it is not part of the hypothesized model and is not tested.

### 2.2 Positive implicit followership theories and innovative behavior

Followers who endorse a positive followership prototype construe the follower role in terms of desirable attributes such as industriousness, enthusiasm, and good citizenship (Sy, 2010). Such positive, self-relevant beliefs are likely to be associated with how employees approach their work in ways that favor innovation. First, employees who hold a positive image of what it means to be a good follower may be more likely to interpret their role expansively, viewing the contribution of ideas and improvements as consistent with, rather than extraneous to, being a good follower. Second, positive prototypes carry affective and motivational connotations—enthusiasm and industriousness imply energy and effort—that may be reflected in the discretionary investment required for innovation. Third, because innovative behavior entails risk and the possibility of failure, employees may benefit from a positive self-relevant frame within which such investment is evaluated as worthwhile and appropriate; a positive followership prototype may supply this frame. Consistent with this reasoning, emerging evidence links positive IFTs and related followership cognitions to favorable, proactive workplace outcomes (Zhang and Wang, 2021; Liang et al., 2022). Followers' positive followership prototype endorsement is therefore expected to be positively related to innovative behavior.

Hypothesis 1 (H1): Followers' positive IFTs are positively related to their innovative behavior.

### 2.3 Positive implicit followership theories and future work self salience

Future work self salience refers to a salient and clear mental representation of the self that one hopes to become in relation to work (Strauss et al., 2012). It is a domain-specific possible self that captures the hopes and aspirations individuals hold for their working lives. Followers' positive IFTs are proposed to be associated with greater future work self salience. A positive followership prototype specifies desirable attributes of the follower role, namely industriousness, enthusiasm, and good citizenship. For an employee who endorses such a prototype, these attributes describe a valued way of being at work and may therefore serve as an aspirational reference point for the working self that the employee hopes to become. A reference point of this kind may make the hoped-for future work self easier to elaborate and to imagine, that is, more salient, coherent, and accessible. Recent evidence also treats future work self salience as a future-oriented motivational resource linked to proactive behavior (He et al., 2022).

This reasoning is consistent with evidence that positive followership beliefs are linked to self-relevant cognitions. For example, positive implicit followership has been shown to relate to newcomers' self-efficacy, indicating that beliefs about the follower role can be associated with beliefs about the self (Zhang and Wang, 2021). Extending this logic from efficacy beliefs to future-oriented self-representations, the more strongly employees endorse a positive followership prototype, the greater their future work self salience is expected to be. A positive prototype provides an aspirational reference point for the future work self, which may make the hoped-for future work self more salient and accessible.

Hypothesis 2 (H2): Followers' positive IFTs are positively related to their future work self salience.

### 2.4 Future work self salience and job crafting

Job crafting refers to the self-initiated, proactive changes that employees make to the task, relational, and cognitive boundaries of their jobs (Wrzesniewski and Dutton, 2001). Within a resource-based operationalization, job crafting includes increasing structural job resources, increasing social job resources, and increasing challenging job demands—forms of approach-oriented crafting through which employees actively shape their work environment (Tims et al., 2012). Future work self salience is proposed to be associated with job crafting as a possible “reason-to” motivational resource (Parker et al., 2010). Employees who hold a clear and valued image of their future working self experience a discrepancy between their current situation and their aspired future, and this discrepancy may provide a reason to take initiative in the present. Because job crafting is a self-initiated, future-oriented behavior through which employees may move toward a desired working self—by acquiring resources, building developmental relationships, and seeking out challenges that promote growth—future work self salience is likely to be linked to more active crafting.

Empirical work supports the link between future work self salience and proactive, self-shaping behavior. Future work self salience has been linked to proactive career behaviors and adaptive, agentic responses at work (Strauss et al., 2012; Zhang et al., 2026; Lin and Nguyen, 2024). Although these studies examine a range of proactive outcomes, they converge on the principle that a salient hoped-for working self may function as a motivational reference associated with proactive investment. Job crafting is a prototypical instance of such investment, in that it represents employees' bottom-up efforts to align their jobs with their preferences, strengths, and aspirations. Greater future work self salience is therefore expected to be associated with more job crafting. Recent work likewise links future work self salience to proactive behavior, although these findings do not directly test job crafting (Voigt and Strauss, 2024). Accordingly, the FWS salience-job crafting link is proposed as a theoretically grounded association; the cited recent studies do not directly establish this specific path.

Hypothesis 3 (H3): Followers' future work self salience is positively related to their job crafting.

The theoretical basis of this link lies in the correspondence between the nature of the future work self salience and the nature of job crafting. Future work self salience reflects a self-generated, forward-looking representation of a hoped-for self (Markus and Nurius, 1986; Strauss et al., 2012), and job crafting is a self-initiated behavior through which employees change their tasks, relationships, and job resources and demands (Wrzesniewski and Dutton, 2001; Tims et al., 2012); both are instances of proactivity, understood as self-initiated, future-focused action aimed at bringing about change (Parker et al., 2010). This correspondence suggests that future work self salience may be linked especially to growth-oriented, approach-focused forms of crafting—acquiring resources, cultivating developmental relationships, and seeking challenges—the promotive dimensions examined here. By contrast, a hoped-for working self is not expected to relate to the reduction of hindering demands in the same way, because that avoidance-oriented form of crafting is less directly instrumental to progress toward an aspirational self. This reasoning informs the decision to treat the promotive dimensions as the focal composite while measuring, but not combining, the reducing-hindering-demands dimension.

### 2.5 Job crafting and innovative behavior

Job crafting is expected to be positively associated with innovative behavior for several interrelated reasons. By increasing structural resources—such as developing skills and seeking autonomy—employees expand their capacity and latitude to experiment with new approaches. By increasing social resources—such as seeking feedback, advice, and support—employees gain access to diverse information and perspectives that stimulate idea generation and facilitate the promotion and implementation of ideas. By seeking out challenging demands, employees place themselves in situations that call for novel solutions and that provide opportunities to learn and to apply new ideas. Through these pathways, job crafting is associated with motivational and resource conditions that support innovation. A meta-analytic synthesis indicates that job crafting is positively associated with a range of favorable work outcomes, including engagement and performance, which are themselves conducive to innovation (Rudolph et al., 2017), and recent studies specifically link job crafting to employees' innovative behavior (Song and Jo, 2023; Miao et al., 2022). Recent longitudinal evidence further indicates that job crafting and innovative work behavior may be dynamically related, rather than strictly unidirectional (Tomas et al., 2023).

It is important to distinguish job crafting from innovative behavior conceptually, given that both are proactive. Job crafting concerns the reshaping of one's own job design—the boundaries, resources, and demands that define how work is carried out—whereas innovative behavior concerns the generation, promotion, and realization of novel ideas that create value beyond the job itself. Job crafting is thus better understood as a proximal, self-directed behavior that alters the conditions of work, while innovative behavior is an outward-facing, idea-oriented outcome. Consistent with this distinction, recent research treats job crafting and innovative behavior as two related but distinct forms of proactive behavior that share some antecedents (Messmann, 2023). This conceptual distinction implies that crafting can be examined as a proximal correlate of innovation rather than being treated as redundant with it, and their empirical distinctiveness is tested explicitly below. Employees' job crafting is expected to be positively related to innovative behavior.

Hypothesis 4 (H4): Followers' job crafting is positively related to their innovative behavior.

Beyond the resource- and challenge-based pathways noted above, job crafting may relate to innovation through changes in the psychological relationship between the employee and the work itself. When employees actively adjust their tasks, relationships, and construal of the job, they come to experience the work as more personally meaningful and more within their control, and this sense of ownership may lower the perceived cost of departing from routine. Innovation is inherently effortful and uncertain, and employees are more willing to bear that effort and uncertainty when they experience ownership of the work. In this way, crafting is associated not only with tangible resources and challenges relevant to innovation, but also with the motivational orientation under which the pursuit of novelty is evaluated as worthwhile. This complementary account supports the expectation that more crafting will be associated with more innovative behavior.

### 2.6 Indirect associations via future work self salience and job crafting

The preceding arguments specify three associations: between positive IFTs and future work self salience (H2), between future work self salience and job crafting (H3), and between job crafting and innovative behavior (H4). If all three hold, positive IFTs should be indirectly associated with innovative behavior via future work self salience and job crafting. The proposed indirect association is internal to the follower and is expected to coexist with, rather than replace, the relational associations documented in prior research. Because positive IFTs may also be associated with innovative behavior through routes not captured by the two intervening variables (for example, direct motivational or affective associations), a direct association between positive IFTs and innovative behavior is expected to remain after the indirect association is accounted for. The hypothesized associations are summarized in Figure 1.

**Figure 1:**
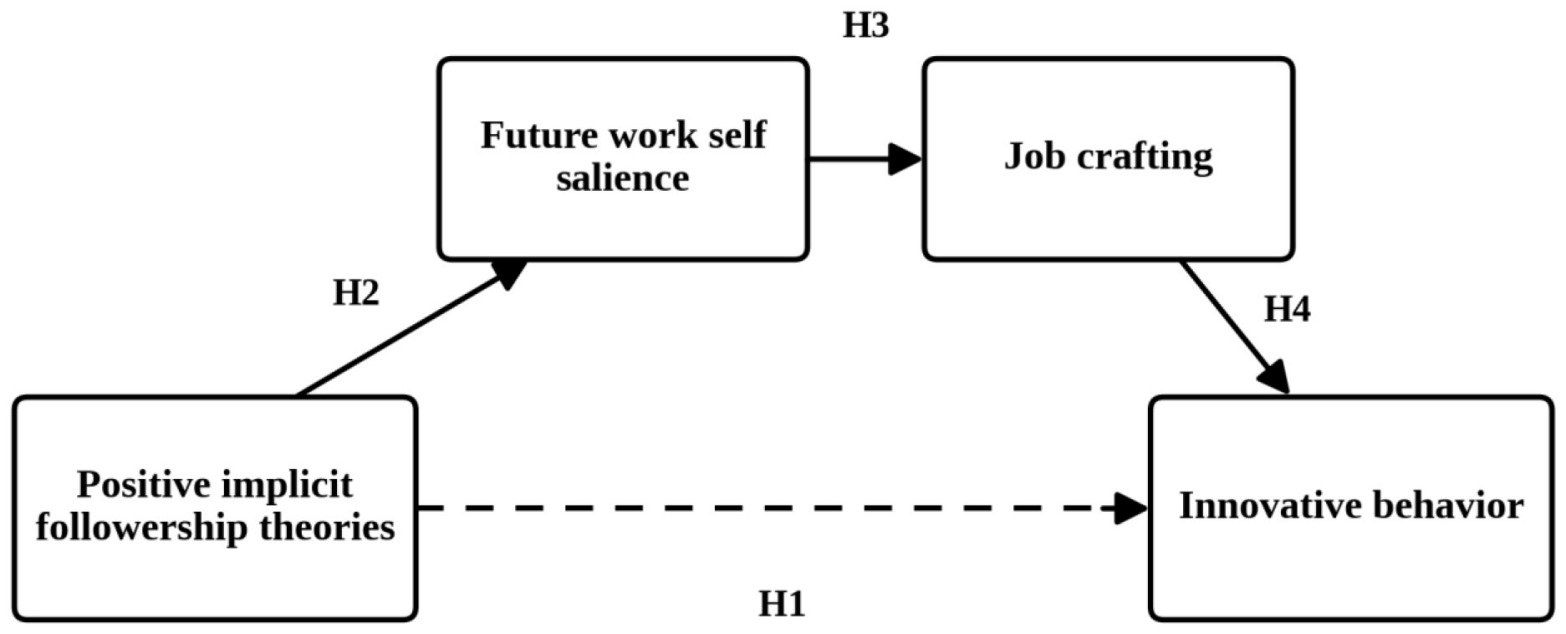
The hypothesized model. Positive implicit followership theories are expected to be positively associated with innovative behavior (H1) and indirectly associated with it via future work self salience and job crafting (H2–H5). Future work self salience and job crafting were both measured at T2.

Hypothesis 5 (H5): Followers' positive IFTs are indirectly associated with their innovative behavior via future work self salience and job crafting (positive IFTs → future work self salience → job crafting → innovative behavior).

## 3 Materials and methods

### 3.1 Participants and procedure

Data were collected from knowledge workers and their direct supervisors in Chinese enterprises located in knowledge-intensive cities, including Shenzhen, Beijing, Shanghai, Hangzhou, Nanjing, and Chengdu, and spanning industries such as information technology and software, electronics and telecommunications, finance, advanced manufacturing and research and development, and professional services. Knowledge workers were selected because innovative behavior is particularly salient for such employees, whose work centers on the generation and application of ideas. To separate the measurement occasions and reduce the risk of common method bias, a three-wave, time-lagged design with matched employee–supervisor dyads was adopted, with an interval of approximately 1 month between adjacent waves. Participation was voluntary, informed consent was obtained, and, to enable matching across waves while protecting confidentiality, each participant was identified only by an anonymized code rather than by identifying information. The three waves of data were collected between February and May 2026. Participants were recruited with the assistance of the human resource departments of the participating enterprises, which distributed the questionnaires to eligible knowledge workers on a voluntary basis. Supervisor identifiers were used to match ratings; the number of employees rated by each supervisor and supervisor-level ICCs were not estimated, and the path models did not include supervisor clustering. The number of participating organizations and potential organization-level clustering were not modeled in the present analysis.

Data were collected in three waves as follows. At Time 1 (T1), employees reported their positive IFTs together with the control variables, namely age, gender, organizational tenure, education, proactive personality, and negative IFTs. At Time 2 (T2), approximately 1 month later, the same employees reported their future work self salience and their job crafting. At Time 3 (T3), approximately 1 month after T2, the employees' direct supervisors rated the employees' innovative behavior. Obtaining the dependent variable from supervisors, and separating the measurement of predictor, mediators, and outcome in time, together provide procedural safeguards against common method bias (Podsakoff et al., 2003).

#### 3.1.1 Data screening and sample retention

The sample-screening procedure was documented step by step. The raw records included 460 responses at T1, 416 at T2, and 353 at T3. Matching the three waves by anonymized ID produced 348 common employee records. Sixty-seven records that failed at least one embedded quality-control item, 13 records that showed invariant straight-line responding across focal scales, and 10 records for which at least one focal composite variable could not be computed were then excluded. The final valid sample therefore comprised 258 employee–supervisor dyads. The focal constructs used to test the hypotheses—positive IFTs, negative IFTs, proactive personality, future work self salience, job crafting, and innovative behavior—had no missing values in this final dataset. No separate statistical comparison between retained and non-retained records was conducted, so selection related to attrition cannot be excluded.

A small number of participants did not report every demographic item (missing rates below 2.5% per variable). To retain all 258 matched dyads in the main analyses, missing values on demographic controls were replaced with the variable mean for continuous variables and the modal category for categorical or ordinal variables; complete-case analyses produced substantively unchanged results. This sample size is adequate for the regression-based path model, which estimates only a small number of parameters, and is consistent with the guideline of roughly five to ten cases per estimated parameter for such models (Bentler and Chou, 1987); as noted below, the ratio is less favorable for the full latent-variable measurement model, whose results are therefore interpreted with corresponding caution. Among valid respondents, 51.2% were men; most were aged 30–39 years (53.5%), and the plurality held a bachelor's degree (43.8%). Full demographic characteristics are reported in Table 1.

**Table 1:** Demographic characteristics of the sample (*N* = 258).

Variable	Category	N	%
Gender	Male	132	51.2
	Female	120	46.5
Age	< = 29	78	30.2
	30–39	138	53.5
	40–49	36	14.0
	> = 50	1	0.4
Tenure	< = 3 years	72	27.9
	4–7 years	108	41.9
	8–15 years	64	24.8
	>15 years	9	3.5
Education	High school or below	13	5.0
	College	57	22.1
	Bachelor	113	43.8
	Master	59	22.9
	Doctor	12	4.7
Position	Staff	153	59.3
	First-line manager	70	27.1
	Middle manager	35	13.6
Industry	IT/Software	41	15.9
	Consulting/Professional services	31	12.0
	Advanced manufacturing/R&D	31	12.0
	Education/Research	29	11.2
	Electronics/Telecommunications	28	10.9
	Finance	25	9.7
	Internet	22	8.5
	Pharmaceuticals/Biotech	18	7.0
	Construction/Engineering design	16	6.2
	Culture/Design	13	5.0
	Not reported	4	1.6

Percentages may not total 100% due to rounding and missing values. Category totals may be below 258 because missing values are not displayed as a separate category.

The Chinese enterprise context is also substantively appropriate for the present model. Contemporary Chinese organizations operate under strong innovation imperatives while retaining relatively hierarchical role structures, which makes the follower role salient; this combination provides a setting in which followers' beliefs about their role, and their agentic responses to those beliefs, are likely to be relevant to innovation. The question of how this context may bound the generalizability of the findings is taken up in the limitations section.

### 3.2 Measures

Most constructs were measured using established scales. The positive IFT measure was adapted with a self-referent framing, and the evidence supporting this adaptation is reported separately below; the reliability and discriminant-validity evidence reported below does not establish the construct validity of this adaptation, as explained in that section. Items originally developed in English were translated into Chinese and independently back-translated to ensure conceptual equivalence (Brislin, 1980). Unless otherwise noted, responses were provided on a five-point Likert scale ranging from 1 (strongly disagree) to 5 (strongly agree). Reliability estimates for all scales are reported below and on the diagonal of Table 3.

Positive implicit followership theories. Employees' endorsement of a positive followership prototype was measured with the nine-item prototype subscale of Sy's (2010) IFT scale (industry, enthusiasm, and good-citizen dimensions). Whereas this subscale was originally used to capture individuals' beliefs about followers in general, it was administered here with a self-referent framing, so as to capture the followership prototype that followers endorse for themselves. Self-referent adaptations of followership prototype and implicit-theory measures have precedent in the literature, in which followers' own prototypes and self-views have been examined as antecedents of their attitudes and behavior (e.g., Zhang and Wang, 2021). To reduce the risk of conceptual overlap with related constructs, proactive personality and negative IFTs were included as control variables, and positive IFTs were specified as a separate factor in the confirmatory factor analysis, in which the six-factor model fit well (see Results). In the present sample, Cronbach's alpha was 0.878. Two qualifications apply to this evidence. First, coefficient alpha indicates only that the nine adapted items were internally consistent, and the CFA results indicate only that responses to these items were empirically distinguishable from responses to the other constructs in this sample. Neither form of evidence establishes that the self-referent items measure the same construct as the original other-referent prototype subscale. Rewording the items to refer to the self changes the referent of the judgment from followers in general to the respondent, and the resulting scores may partly reflect self-descriptions of follower-relevant attributes rather than an implicit theory about followers. Second, no evidence of content, convergent, or criterion validity for the self-referent version is available in the present study. The construct measured here is therefore best described as followers' self-endorsed positive followership prototype, and the results involving this variable should be interpreted with this measurement limitation in mind.

Future work self salience. Future work self salience was measured with the five-item scale developed by (Strauss et al. 2012), which assesses the clarity and accessibility of respondents' hoped-for working selves. A sample item asks respondents to indicate how easily they can imagine their future work self. Cronbach's alpha was 0.836. This measure therefore captures the salience, clarity, and accessibility of the hoped-for future work self rather than the specific content or positivity of that future self.

Job crafting. Job crafting was measured with the scale of (Tims et al. 2012). Consistent with the theoretical focus on approach-oriented crafting, the focal composite comprised the three promotive dimensions—increasing structural job resources, increasing social job resources, and increasing challenging job demands. Cronbach's alpha for the focal composite was 0.909. The reducing-hindering-demands dimension, which is conceptually and empirically distinct from the promotive dimensions and is often avoidance-oriented, was measured but retained separately and was not combined into the focal composite; it was not the focus of the present hypotheses.

Innovative behavior. Employees' innovative behavior was rated by their direct supervisors using the six-item scale of (Scott and Bruce 1994), which captures idea generation, idea promotion, and idea realization. Using supervisor ratings for the dependent variable reduces reliance on common-source self-reports. Cronbach's alpha was 0.859.

Control variables. Age, gender, organizational tenure, and education were controlled, because these demographic factors may relate to future work self salience, job crafting, or innovative behavior. In addition, because proactive personality and negative IFTs may plausibly relate to both the mediators and the outcome, and could therefore offer alternative explanations, proactive personality was controlled, measured with the scale of (Seibert et al. 2001) (Cronbach's alpha = 0.875), as were negative IFTs, measured with the antiprototype subscale of (Sy 2010) (Cronbach's alpha = 0.828). Controlling for these variables provides a conservative test of the incremental role of followers' positive IFTs.

### 3.3 Analytical strategy

Analyses proceeded in four steps. First, confirmatory factor analysis (CFA) was conducted after replacing the approximately 1.03% missing item-level values by item means, using the full matched sample (*N* = 258), to evaluate the measurement model and, in particular, the discriminant validity of the focal constructs, comparing the hypothesized model with a series of more parsimonious alternative models in which conceptually related constructs were merged (Anderson and Gerbing, 1988). Second, common method variance was assessed using Harman's single-factor test as an auxiliary check, alongside the procedural safeguards built into the design. Third, the hypothesized direct and indirect effects were estimated using a regression-based path-analytic approach with observed composite scores, corresponding to Hayes' PROCESS Model 6 (a serial mediation model in which future work self salience is entered before job crafting), rather than a full latent-variable structural equation model. The observed-variable approach was chosen because the analytic focus was on bootstrap inference for the specific and two-mediator indirect effects, and because the ratio of sample size to the number of parameters in a full latent mediation model incorporating all indicators would have been unfavorable; latent-variable CFA was nonetheless used beforehand to evaluate measurement quality and discriminant validity. Fourth, the indirect effects specified in H5 were estimated using bias-corrected bootstrapping with 5,000 resamples and 95% confidence intervals (CIs); consistent with contemporary recommendations, an indirect effect was deemed statistically significant when its CI excluded zero (Preacher and Hayes, 2008; Taylor et al., 2008). The main path analyses used the full matched sample (*N* = 258) after replacement of missing demographic controls, and the same models were rerun using the 238 cases with complete data on all demographic control variables as a robustness check. All analyses controlled for age, gender, tenure, education, proactive personality, and negative IFTs. Because the structural relations were estimated with observed-variable path analysis rather than a latent structural model, model fit indices are reported for the CFA measurement model only and are not applicable to the path model. Following statistical convention, the terms “effect,” “direct effect,” “indirect effect,” and “mediation” are used to describe the decomposition of associations in the regression model; they do not imply causal influence or temporal precedence.

Four features of the analytical approach should be noted. Bias-corrected bootstrapping was used rather than the causal-steps or Sobel approaches because bootstrapping makes no assumption that the indirect effect is normally distributed and provides more appropriate inference for the products of coefficients that define the two-mediator indirect effect. The specific indirect effects for each single-mediator path were estimated and reported in addition to the two-mediator indirect effect, because doing so allows the two-mediator indirect effect to be distinguished from the two single-mediator indirect effects. The direct effect was retained in the model rather than assuming full mediation, so that partial mediation could be detected if present. Finally, the same set of controls was carried through all structural analyses, so that the direct and indirect effects were estimated with a common, conservative set of covariates that accounts for demographic factors as well as proactive personality and negative IFTs. Collectively, these choices reflect current recommendations for estimating indirect effects in models with two intervening variables and support a cautious interpretation of the results.

## 4 Results

### 4.1 Common method variance

The primary safeguards against common method bias were procedural: the predictor, the intervening variables, and the outcome were measured at different waves, and innovative behavior was rated by supervisors; the two intervening variables, however, were self-reported at the same wave. As an auxiliary check, Harman's single-factor model fit the data poorly (χ^2^ = 4,136.33, *df* = 1,377, χ^2^*/df* = 3.004, CFI = 0.414, TLI = 0.391, RMSEA = 0.088). Because this test is limited in its ability to detect common method variance, the result is interpreted alongside the procedural safeguards rather than as evidence that such bias is absent.

### 4.2 Confirmatory factor analysis and validity

The measurement model was evaluated using CFA after item-mean imputation for the approximately 1.03% missing item-level values. The six-factor model was estimated using the full matched sample (N = 258) and fit the data well (Table 2; χ^2^ = 1,455.45, *df* = 1,362, χ^2^*/df* = 1.069, CFI = 0.980, TLI = 0.979, RMSEA = 0.016). Fit was poorer when job crafting and innovative behavior were combined (χ^2^ = 1,917.52, *df* = 1,367, χ^2^*/df* = 1.403, CFI = 0.883, TLI = 0.878, RMSEA = 0.040), or when proactive personality and future work self salience were combined (χ^2^ = 1,858.60, *df* = 1,367, χ^2^*/df* = 1.360, CFI = 0.896, TLI = 0.891, RMSEA = 0.037). The one-factor model also fit poorly (χ^2^ = 4,136.33, *df* = 1,377, χ^2^*/df* = 3.004, CFI = 0.414, TLI = 0.391, RMSEA = 0.088).

**Table 2:** Confirmatory factor analysis and model comparison (*N* = 258).

Model	chi-square	*df*	chi-square/*df*	CFI	TLI	RMSEA
Six-factor (hypothesized)	1455.45	1362	1.069	0.980	0.979	0.016
JC and IB merged	1917.52	1367	1.403	0.883	0.878	0.040
PP and FWS merged	1858.60	1367	1.360	0.896	0.891	0.037
One-factor (Harman)	4136.33	1377	3.004	0.414	0.391	0.088

IFT, implicit followership theories; FWS, future work self salience; JC, job crafting; IB, innovative behavior; PP, proactive personality. The hypothesized six-factor model includes positive IFTs, negative IFTs, PP, FWS, JC, and IB.

The confirmatory factor analysis results bear on the interpretation of the substantive findings. Because job crafting and innovative behavior are both proactive, self-initiated behaviors, a reasonable concern is that they might be empirically indistinguishable, in which case the H4 relationship would be inflated by construct overlap rather than an association between distinct constructs. The deterioration in fit when the two constructs were forced onto a single factor indicates that the two constructs were empirically distinct. The use of a different source for the outcome—supervisor ratings—further reduces the plausibility of a common-factor artifact. Together with the poor fit of the one-factor model and the design-based separation of measurement, these results indicate that the focal constructs were empirically distinguishable in this sample and make a single common-factor explanation less plausible. They do not establish that the constructs are conceptually distinct, and they do not rule out shared method variance between future work self salience and job crafting, which were both self-reported at T2. For the self-referent positive IFT measure, this evidence concerns distinctiveness only; it does not address whether the adapted items capture the intended construct (see Measures). Given the ratio of sample size to the number of estimated parameters, these measurement-model results should be interpreted with appropriate caution regarding parameter stability, and replication with larger samples would be valuable.

### 4.3 Descriptive statistics and correlations

Means, standard deviations, reliabilities, and zero-order correlations among the study variables are reported in Table 3. The pattern of correlations was consistent with the hypothesized relationships. Positive IFTs were positively correlated with future work self salience (*r* = 0.32, *p* < 0.01), job crafting (*r* = 0.28, *p* < 0.01), and innovative behavior (*r* = 0.33, *p* < 0.01). Future work self salience was positively correlated with job crafting (*r* = 0.37, *p* < 0.01), and job crafting was positively correlated with innovative behavior (*r* = 0.38, *p* < 0.01). Negative IFTs were weakly and generally negatively associated with the focal variables, and proactive personality showed modest positive correlations with several variables, supporting the inclusion of these constructs as controls. The bivariate correlations were consistent with the hypothesized associations.

**Table 3:** Means, standard deviations, reliabilities, and correlations.

Variable	M	SD	1	2	3	4	5	6
1 Positive IFT	3.61	0.56	(0.88)					
2 Future work self salience	3.38	0.66	0.32	(0.84)				
3 Job crafting	3.33	0.54	0.28	0.37	(0.91)			
4 Innovative behavior	3.33	0.66	0.33	0.23	0.38	(0.86)		
5 Negative IFT	2.37	0.53	−0.01	0.05	−0.05	−0.09	(0.83)	
6 Proactive personality	3.46	0.54	0.19	0.30	0.08	0.27	0.03	(0.88)

*N* = 258. Cronbach's alpha appears on the diagonal in parentheses. Correlations with |*r*| >= 0.13 are significant at *p* < 0.05; |*r*| >= 0.17 at *p* < 0.01.

### 4.4 Hypothesis testing: direct effects

The direct effects specified in H1–H4 were tested using the regression-based path model, with results summarized in Table 4 and depicted in Figure 2. Positive IFTs had a significant positive total effect on innovative behavior (β = 0.293, *p* < 0.001), supporting H1. Consistent with H2, positive IFTs were positively associated with future work self salience (β = 0.270, *p* < 0.001). Consistent with H3, future work self salience was positively associated with job crafting (β = 0.323, *p* < 0.001). Consistent with H4, job crafting was positively associated with innovative behavior (β = 0.311, *p* < 0.001). When the mediators were included in the model, the direct effect of positive IFTs on innovative behavior remained significant (β = 0.208, *p* < 0.001), indicating that the direct association was not fully accounted for by the two intervening variables (partial mediation in the statistical sense). Two additional component paths of the model were also estimated: positive IFTs were significantly associated with job crafting (β = 0.186, *SE* = 0.060, *t* = 3.08, *p* = 0.002), whereas the direct path from future work self salience to innovative behavior was non-significant (β = −0.000, *SE* = 0.063, *t* = −0.00, *p* = 0.998); these component paths are consistent with the specific indirect effects reported below.

**Table 4:** Path model: standardized direct effects.

Path	Hypothesis	beta	SE	*t*	*p*	Result
Positive IFT -> IB (total)	H1	0.293	0.059	4.96	< 0.001	Supported
Positive IFT -> FWS salience	H2	0.270	0.059	4.56	< 0.001	Supported
FWS salience -> JC	H3	0.323	0.062	5.20	< 0.001	Supported
JC -> IB	H4	0.311	0.061	5.08	< 0.001	Supported
Positive IFT -> JC	-	0.186	0.060	3.08	0.002	Significant
FWS salience -> IB	-	−0.000	0.063	−0.00	0.998	n.s.
Positive IFT -> IB (direct)	-	0.208	0.060	3.49	< 0.001	Direct association retained

IFT, implicit followership theories; FWS, future work self salience; JC, job crafting; IB, innovative behavior. All analyses control for age, gender, tenure, education, proactive personality, and negative IFTs. Positive IFT → JC and FWS salience → IB are additional component paths of the model.

**Figure 2:**
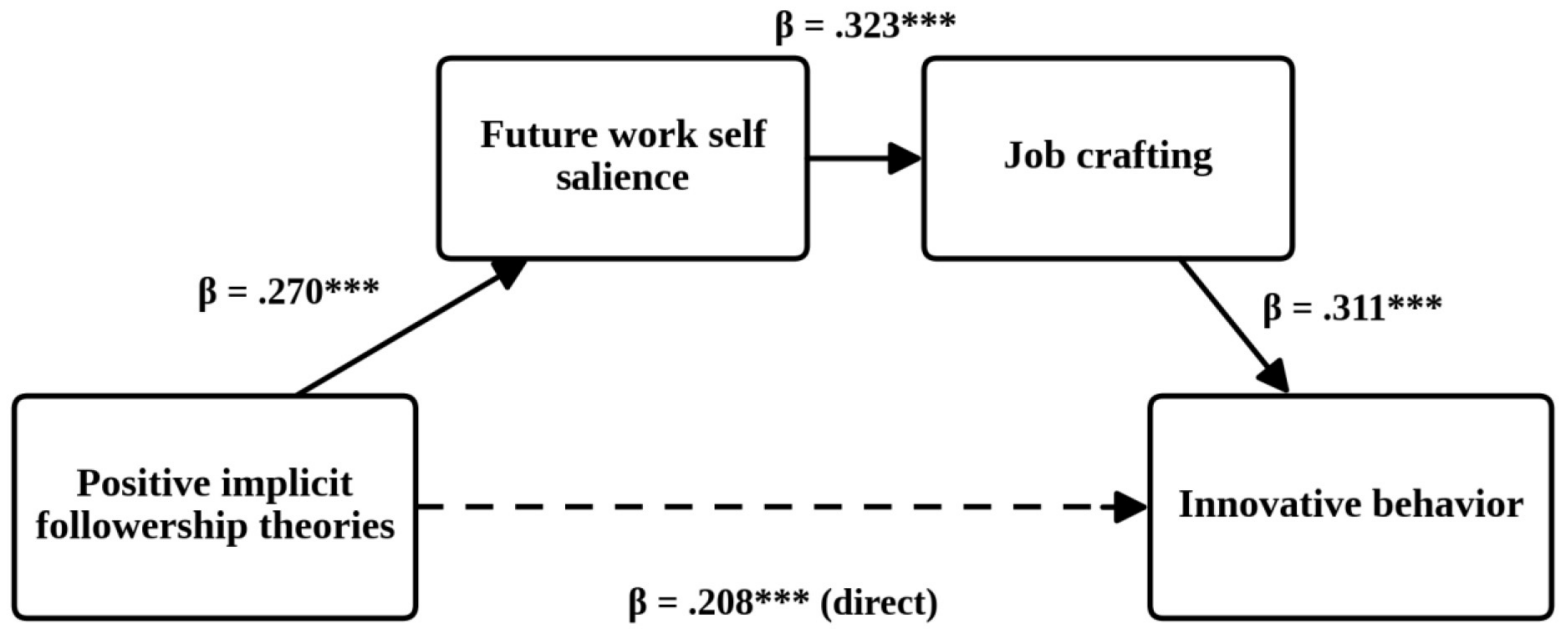
Standardized path estimates of the regression-based path model. ***p < 0.001.

### 4.5 Indirect associations (H5)

The indirect effects specified in H5 were estimated with bias-corrected bootstrapping (5,000 resamples); results are reported in Table 5 and Figure 3. The indirect effect via future work self salience and job crafting was statistically significant (effect = 0.027, 95% CI [0.011, 0.048]), consistent with H5. Because future work self salience and job crafting were both measured at T2, this estimate provides no evidence about the temporal ordering of the two variables. The indirect effect via job crafting alone was also significant (effect = 0.058, 95% CI [0.021, 0.105]), whereas the indirect effect via future work self salience alone was not (effect = −0.000, 95% CI [−0.033, 0.035]). Thus, although future work self salience was correlated with innovative behavior at the bivariate level (*r* =0.23), it showed no unique association with innovative behavior once job crafting and the covariates were included in the model. Together with the significant direct association reported above, these results indicate that positive IFTs were both directly and indirectly associated with innovative behavior.

**Table 5:** Bootstrap estimates of direct and indirect effects (PROCESS Model 6; 5,000 resamples).

Effect	Path	Estimate	95% LLCI	95% ULCI	Significance
Total	Positive IFT -> IB	0.293	-	-	-
Direct	Positive IFT -> IB	0.208	-	-	-
Indirect	Positive IFT -> FWS salience -> IB	−0.000	−0.033	0.035	n.s.
Indirect	Positive IFT -> JC -> IB	0.058	0.021	0.105	Significant
Indirect (two mediators)	Positive IFT -> FWS salience -> JC -> IB	0.027	0.011	0.048	Significant

IFT, implicit followership theories; FWS, future work self salience; JC, job crafting; IB, innovative behavior. LLCI/ULCI, lower/upper limit of the 95% bias-corrected confidence interval. An indirect effect is significant when the CI excludes zero. FWS salience and JC were both measured at T2. As a robustness check, the path and bootstrap analyses were repeated using the 238 cases with complete data on all demographic control variables. The pattern of findings was substantively unchanged, and the two-mediator indirect effect remained significant in the complete-case analysis (effect = 0.026, 95% CI [0.010, 0.049]).

**Figure 3:**
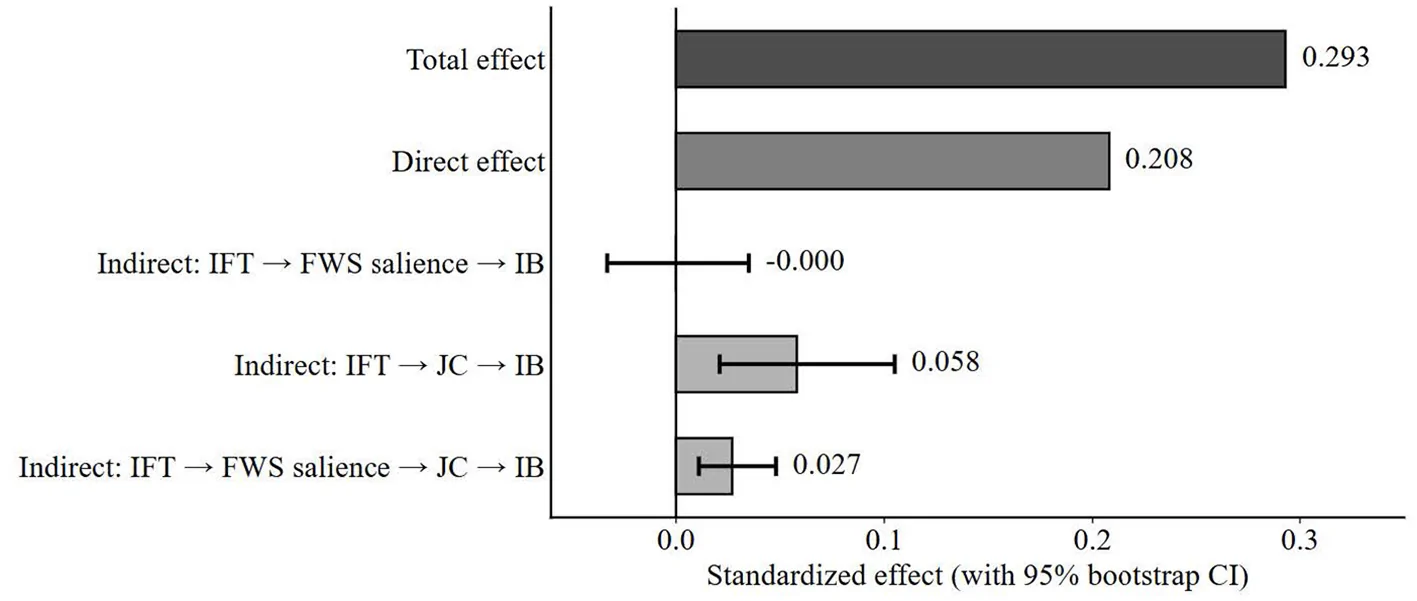
Decomposition of the total, direct, and indirect effects on innovative behavior, with 95% bias-corrected bootstrap confidence intervals.

The two-mediator indirect effect was small in absolute terms. Indirect effects that pass through two intervening variables are typically smaller than single-mediator indirect effects because they are products of three coefficients; the estimate of 0.027 should therefore be interpreted as a statistically reliable but modest indirect association in this sample. The non-significant indirect effect via future work self salience alone, together with the significant indirect effects via job crafting, indicates that the association between future work self salience and innovative behavior in this sample was accounted for by job crafting. The significant direct association indicates that the two intervening variables did not account for the full association between positive IFTs and innovative behavior.

### 4.6 Robustness checks and model diagnostics

Several additional analyses were conducted to assess the robustness of the findings and to provide model diagnostics. First, multicollinearity was low: in the most fully specified model, variance inflation factors for all predictors were low in both the full sample and the complete-case sample (all VIFs ≤ 1.37), well below conventional thresholds. Second, in the full sample, the models explained 16.7% of the variance in future work self salience (adjusted *R*^2^ =0.143), 19.8% in job crafting (adjusted *R*^2^ =0.173), and 25.4% in innovative behavior (adjusted *R*^2^ =0.227).

Third, because the main analyses replaced a small number of missing demographic controls, all path and bootstrap analyses were re-estimated using the 238 cases with complete data on all demographic control variables; the direction and significance of every focal path were unchanged, and the two-mediator indirect effect remained significant (effect = 0.026, 95% CI [0.010, 0.049]). Fourth, to address the concern that the focal job-crafting composite aggregated three promotive dimensions, the separately measured reducing-hindering-demands dimension was examined in two ways. When this dimension was added as an additional control, the two-mediator indirect effect remained significant (full sample: effect = 0.026, 95% CI [0.012, 0.047]; complete cases: effect = 0.026, 95% CI [0.010, 0.047]), all focal path coefficients changed by no more than 0.005, and the reducing-hindering-demands dimension was itself unrelated to innovative behavior (β = 0.07, *p* = 0.24). When it was instead combined with the three promotive dimensions into a four-dimension composite (which correlated 0.94 with the focal composite), the two-mediator indirect effect again remained significant (full sample: effect = 0.029, 95% CI [0.013, 0.052]; complete cases: effect = 0.028, 95% CI [0.011, 0.050]). The substantive conclusions were therefore not sensitive to how job crafting was operationalized.

## 5 Discussion

Adopting a follower-centric perspective and integrating possible-selves theory with the model of proactive motivation, the present study examined whether followers' own positive IFTs are directly and indirectly associated with their innovative behavior. Three findings emerged. First, followers' positive IFTs were positively associated with supervisor-rated innovative behavior 2 months later. Second, positive IFTs were indirectly associated with innovative behavior via future work self salience and job crafting, and the direct association remained significant when the two intervening variables were included. Third, the indirect association was carried by the paths involving job crafting; future work self salience alone was not indirectly associated with innovative behavior. Because future work self salience and job crafting were measured at the same wave, the findings do not establish their temporal order, nor causal relations among the constructs.

The overall pattern can be summarized as follows. Employees who more strongly endorsed a positive followership prototype reported greater future work self salience and more job crafting, and they were rated by their supervisors as more innovative; the associations among these variables were consistent with an indirect association via self-concept and proactive work behavior. This interpretation locates the relevant correlates in followers' self-concept and agentic behavior, in addition to the interpersonal expectation processes emphasized in prior research, which were not measured here, and it points to cognitions and behaviors that individuals and organizations may plausibly influence.

### 5.1 Theoretical implications

This study makes three theoretical contributions. First, by positioning followers' own positive IFTs as a construct associated with innovative behavior, it advances a follower-centric account that complements the predominantly leader-centered literature on followership and IFTs. Whereas prior work has largely examined how leaders' beliefs about followers, or leaders' behaviors, relate to follower outcomes, the present findings indicate that followers' own prototypes are associated with innovation, thereby treating followers as agentic actors and responding to calls for research on proactivity and innovation from a followership perspective (Uhl-Bien et al., 2014; Yalçinyigit and Karaçay, 2025).

This contribution also concerns the conceptual status of IFTs themselves. Prior IFT research has mainly examined the IFTs held by leaders or managers as beliefs about their followers, which become relevant to follower outcomes when leaders act on these beliefs toward the perceived follower, for example in the quality of the exchange relationships they form (Sy, 2010; Epitropaki et al., 2013; Liang et al., 2022). The following interpretation is offered tentatively, because, as noted in the Measures section, the self-referent measure has not been shown to capture the same construct as the original IFT measure. The present results are consistent with the possibility that IFTs can also be self-directed: when the follower is both the holder of the prototype and its referent, the belief may be associated with self-definition rather than serving only as a basis for judging others. Whether such a self-definitional process occurs was not examined here. If this possibility is supported with validated measures, a self-directed use of IFTs would extend the scope of the construct and would suggest that the consequences of implicit theories depend not only on their content but also on whom they concern. Because most employees occupy follower roles in at least some of their work relationships, such self-directed prototypes may be a widespread and under-examined correlate of proactive, innovative behavior. This interpretation is consistent with prior work that has examined leaders' IFTs and relational outcomes, as well as recent reviews that distinguish follower-centered and self-relevant followership perspectives (Liang et al., 2022; Matshoba-Ramuedzisi et al., 2022; Ribbat et al., 2024).

Second, by examining future work self salience and job crafting as intervening variables, the study develops an internal, self-concept-based agentic account that complements, rather than competes with, the relational pathways emphasized in prior IFT research. Whereas earlier work has explained the link between IFTs and innovation through the quality of the leader–follower relationship (Liang et al., 2022), the present findings indicate that positive prototypes are also associated with innovation via variables that are internal to the follower, namely the salience of employees' future work selves and their job crafting. This complements relational accounts and suggests that IFTs may be associated with outcomes through multiple, coexisting pathways.

Third, the study integrates possible-selves theory with the model of proactive motivation and, in doing so, specifies how role-relevant beliefs may be associated with agentic behavior. The results are consistent with the view that future work self salience may function as a “reason-to” motivational resource for proactive crafting, and that crafting is associated with innovation (Markus and Nurius, 1986; Parker et al., 2010). The observation that future work self salience showed no unique association with innovation once job crafting was taken into account is consistent with the theoretical claim that hoped-for selves are associated with distal outcomes in conjunction with proximal proactive behavior.

More broadly, the pattern of indirect effects has a theoretical implication. That the indirect effects involving job crafting were significant while the indirect effect via future work self salience alone was not suggests that, in this sample, the association between future work self salience and innovation was accounted for by job crafting. The pattern is consistent with job crafting, as a concrete reshaping of tasks, relationships, and challenges, connecting future-oriented self-cognition with innovation-relevant conditions. For possible-selves accounts, this suggests that models of distal outcomes may benefit from specifying the proactive behaviors with which future-oriented self-representations co-occur. The results are also relevant to a recurring question in the proactivity literature about how cognitive and motivational states are associated with work outcomes, and they identify job crafting as one behavioral correlate that merits further study in the domain of innovation.

### 5.2 Practical implications

Several tentative practical implications follow from the findings, subject to the limitations noted below. First, because followers' own positive prototypes were associated with innovation, organizations may benefit from cultivating positive, agentic conceptions of the follower role—for example, through onboarding, communication, and development practices that frame effective followership in terms of industriousness, enthusiasm, and constructive citizenship rather than compliance alone. Second, because future work self salience was associated with job crafting, developmental practices that help employees articulate clear images of their working futures—such as career-visioning exercises and developmental conversations—may be relevant to the associations observed here (Zhang et al., 2026). Third, because job crafting was the proximal behavioral correlate of innovation in the model, organizations may support innovation-related behavior by enabling and encouraging job crafting, for instance by granting autonomy, providing access to social resources and feedback, and creating opportunities for employees to take on challenging assignments (Miao et al., 2022).

These implications are best treated as mutually reinforcing possibilities rather than stand-alone prescriptions. Because job crafting was the intervening variable most closely associated with innovation in this model, initiatives directed at beliefs and future selves could be combined with conditions that permit crafting; whether such combinations are more effective than either alone was not tested and remains a question for intervention research. Managers could therefore combine role-framing and career-development practices, such as framing the follower role positively and helping employees articulate valued working futures, with structural enablers such as autonomy, developmental feedback, and access to challenging work. A further implication concerns selection and socialization: to the extent that positive followership prototypes can be strengthened through experience and framing, organizations may treat them as amenable to development and may cultivate them as part of supporting innovation-related behavior. Whether these implications generalize beyond the present setting is an empirical question, and their specific implementation would in any case need to be adapted to organizational culture and context.

### 5.3 Limitations and future research directions

Several limitations should be acknowledged. First, although innovative behavior was rated by supervisors and the predictor and mediators were separated in time, positive IFTs, future work self salience, and job crafting were all self-reported, and the design was time-lagged and non-experimental; the findings are therefore correlational and do not support causal inference. In addition, future work self salience and job crafting were both measured at T2, so the design provides no evidence about their temporal ordering; the two-mediator indirect effect is equally consistent with the reverse order or with a concurrent association between the two variables. Because both variables were also reported by the same respondents at the same wave, their association, and therefore the H3 path and the two-mediator indirect effect, may be inflated by shared method variance. Future research could employ cross-lagged panel designs, experience-sampling methods, or experimental manipulations of positive prototypes and future work self salience to strengthen causal evidence (Strauss et al., 2012; Yu and Feng, 2024). Second, the sample was drawn from knowledge workers in a single national context, which may limit generalizability to other occupations and cultures; replication across settings would be valuable. Third, the present model did not incorporate boundary conditions. Future work could examine moderators—such as job autonomy or organizational innovation climate—that may strengthen or attenuate the proposed associations, and could incorporate additional dimensions or forms of job crafting, including the reducing-hindering-demands dimension that was measured but not included in the focal composite (Rudolph et al., 2017). Finally, future research could investigate whether the proposed associations generalize to other proactive outcomes, such as voice or taking charge, thereby testing the breadth of the follower-centric, self-concept-based account developed here.

Another limitation concerns measurement and data structure. The positive IFT measure was adapted with a self-referent framing. The internal consistency and discriminant validity reported for this measure do not establish its construct validity: they show that the items cohered and were distinguishable from the other measures, not that they capture the same construct as the original other-referent prototype subscale. It is possible that the adapted items partly measure self-perceived follower attributes rather than an implicit theory about followers, in which case the associations reported here would concern a related but different construct. Establishing the construct validity of the self-referent version, for example by examining its convergence with the original version, its relations with follower identification and self-concept measures, and its nomological network, is a necessary step before the present findings can be interpreted with confidence. In addition, the analyses did not model potential supervisor-level nesting in innovative-behavior ratings. Future studies should report the number of employees rated by each supervisor, estimate intraclass correlations, and, where necessary, use clustered standard errors or multilevel models. Finally, although the main analyses retained the full matched sample by replacing a small number of missing demographic controls and the complete-case analyses produced substantively consistent results, future research would benefit from preregistered missing-data rules and larger samples. Attrition from T1 to the final matched sample was also substantial (460 to 258), and because retained and non-retained participants were not compared statistically, the possibility that the final sample differs systematically from the initial respondents cannot be ruled out.

Three further directions for research follow from the present study. First, the self-categorization account described as background in the theory section implies that follower identification could condition the association between positive IFTs and future work self salience. Measuring follower identification and testing it as a first-stage moderator would allow this account, which the present study did not test, to be examined directly. Second, although positive and negative IFTs were treated as distinct and the latter was controlled, future work could examine whether negative prototypes are associated with the self-concept and proactive behavior in their own, possibly non-mirror-image, ways, since the absence of a positive prototype is not necessarily equivalent to the presence of a negative one. Third, the two-mediator indirect effect was modest, as is typical of indirect effects that are products of three coefficients. Replication with larger samples and with designs capable of detecting and distinguishing small effects would help establish the robustness and practical importance of the indirect association. Pursuing these directions would refine understanding of the conditions under which followers' beliefs about their role are indirectly associated with innovation.

## 6 Conclusion

Using three-wave data from 258 employee–supervisor dyads, the present study found that followers' own positive implicit followership theories were positively associated with supervisor-rated innovative behavior, and that this association was partly accounted for by an indirect association via future work self salience and job crafting, with job crafting as the variable most closely associated with innovative behavior. These findings are consistent with a follower-centric account in which followers' beliefs about their role are associated with their self-concept and proactive work behavior, complementing the relational accounts that have dominated research on implicit followership theories. The findings are correlational: the two intervening variables were measured at the same wave, positive IFTs were assessed with a self-referent adaptation whose construct validity remains to be established, and causal and temporal relations among the constructs were not tested. Within these limits, the study indicates that followers' own positive followership beliefs are a correlate of innovative behavior that merits further investigation with designs capable of examining ordering and causality.

## Data Availability

The raw data supporting the conclusions of this article will be made available by the authors, without undue reservation.
